# Supplementary material for: Resolving the full spectrum of human genome variation using Linked-Reads
Source: Genome Res. 2019 Apr;29(4):635–45. doi: 10.1101/gr.234443.118 (PMC6442396; doi:10.1101/gr.234443.118)
Supplement: Supplemental Material [file supp_gr.234443.118_Supplemental_longranger-2.2.zip › longranger-2.2.tar.bz2!/lr2.2-public/lib/stan/cmdstan/stan/doc/api/html/dir_d06f81619dd9bcc28d70dd2c97937727.html]

Stan: src/stan/math/functions Directory Reference


|  |  |
| --- | --- |
|  | Stan  2.5.0  probability, sampling & optimization |


- Main Page
- Related Pages
- Namespaces
- Classes
- Files

All Classes Namespaces Files Functions Variables Typedefs Enumerations Enumerator Friends Macros Pages

- src
- stan
- math
- functions

functions Directory Reference

|  |  |
| --- | --- |
| Files | |
| file | abs.hpp [code] |
|  | |
| file | as\_bool.hpp [code] |
|  | |
| file | bessel\_first\_kind.hpp [code] |
|  | |
| file | bessel\_second\_kind.hpp [code] |
|  | |
| file | binary\_log\_loss.hpp [code] |
|  | |
| file | binomial\_coefficient\_log.hpp [code] |
|  | |
| file | digamma.hpp [code] |
|  | |
| file | dist.hpp [code] |
|  | |
| file | divide.hpp [code] |
|  | |
| file | dot.hpp [code] |
|  | |
| file | dot\_self.hpp [code] |
|  | |
| file | exp2.hpp [code] |
|  | |
| file | falling\_factorial.hpp [code] |
|  | |
| file | fdim.hpp [code] |
|  | |
| file | gamma\_p.hpp [code] |
|  | |
| file | gamma\_q.hpp [code] |
|  | |
| file | ibeta.hpp [code] |
|  | |
| file | if\_else.hpp [code] |
|  | |
| file | int\_step.hpp [code] |
|  | |
| file | inv.hpp [code] |
|  | |
| file | inv\_cloglog.hpp [code] |
|  | |
| file | inv\_logit.hpp [code] |
|  | |
| file | inv\_sqrt.hpp [code] |
|  | |
| file | inv\_square.hpp [code] |
|  | |
| file | inverse\_softmax.hpp [code] |
|  | |
| file | is\_inf.hpp [code] |
|  | |
| file | is\_nan.hpp [code] |
|  | |
| file | is\_uninitialized.hpp [code] |
|  | |
| file | lbeta.hpp [code] |
|  | |
| file | lgamma.hpp [code] |
|  | |
| file | lmgamma.hpp [code] |
|  | |
| file | log1m.hpp [code] |
|  | |
| file | log1m\_exp.hpp [code] |
|  | |
| file | log1m\_inv\_logit.hpp [code] |
|  | |
| file | log1p.hpp [code] |
|  | |
| file | log1p\_exp.hpp [code] |
|  | |
| file | log2.hpp [code] |
|  | |
| file | log\_diff\_exp.hpp [code] |
|  | |
| file | log\_falling\_factorial.hpp [code] |
|  | |
| file | log\_inv\_logit.hpp [code] |
|  | |
| file | log\_rising\_factorial.hpp [code] |
|  | |
| file | log\_sum\_exp.hpp [code] |
|  | |
| file | logical\_and.hpp [code] |
|  | |
| file | logical\_eq.hpp [code] |
|  | |
| file | logical\_gt.hpp [code] |
|  | |
| file | logical\_gte.hpp [code] |
|  | |
| file | logical\_lt.hpp [code] |
|  | |
| file | logical\_lte.hpp [code] |
|  | |
| file | logical\_negation.hpp [code] |
|  | |
| file | logical\_neq.hpp [code] |
|  | |
| file | logical\_or.hpp [code] |
|  | |
| file | logit.hpp [code] |
|  | |
| file | max.hpp [code] |
|  | |
| file | min.hpp [code] |
|  | |
| file | modified\_bessel\_first\_kind.hpp [code] |
|  | |
| file | modified\_bessel\_second\_kind.hpp [code] |
|  | |
| file | modulus.hpp [code] |
|  | |
| file | multiply\_log.hpp [code] |
|  | |
| file | owens\_t.hpp [code] |
|  | |
| file | Phi.hpp [code] |
|  | |
| file | Phi\_approx.hpp [code] |
|  | |
| file | primitive\_value.hpp [code] |
|  | |
| file | promote\_scalar.hpp [code] |
|  | |
| file | promote\_scalar\_type.hpp [code] |
|  | |
| file | rising\_factorial.hpp [code] |
|  | |
| file | scaled\_add.hpp [code] |
|  | |
| file | sign.hpp [code] |
|  | |
| file | square.hpp [code] |
|  | |
| file | step.hpp [code] |
|  | |
| file | sub.hpp [code] |
|  | |
| file | sum.hpp [code] |
|  | |
| file | trigamma.hpp [code] |
|  | |
| file | value\_of.hpp [code] |
|  | |


---

[ Stan Home Page ]

*© 2011–2014,
Stan Development Team.*
